# A Systems Approach to Evaluate One Health Initiatives

**DOI:** 10.3389/fvets.2018.00023

**Published:** 2018-03-09

**Authors:** Simon R. Rüegg, Liza Rosenbaum Nielsen, Sandra C. Buttigieg, Mijalche Santa, Maurizio Aragrande, Massimo Canali, Timothy Ehlinger, Ilias Chantziaras, Elena Boriani, Miroslav Radeski, Mieghan Bruce, Kevin Queenan, Barbara Häsler

**Affiliations:** ^1^Vetsuisse Faculty, University of Zurich, Zurich, Switzerland; ^2^Faculty of Health and Medical Sciences, University of Copenhagen, Copenhagen, Denmark; ^3^Faculty of Health Sciences, University of Malta, Msida, Malta; ^4^Faculty of Economics—Skopje, Saints Cyril and Methodius University of Skopje, Skopje, Macedonia; ^5^Department of Agricultural and Food Sciences, University of Bologna, Bologna, Italy; ^6^Center for Global Health Equity, University of Wisconsin Milwaukee, Milwaukee, WI, United States; ^7^Faculty of Veterinary Medicine, Ghent University, Ghent, Belgium; ^8^Global Decision Support Initiative (GDSI), Technical University of Denmark, Kongens Lyngby, Denmark; ^9^National Food Institute, Technical University of Denmark, Kongens Lyngby, Denmark; ^10^Faculty of Veterinary Medicine, Saints Cyril and Methodius University of Skopje, Skopje, Macedonia; ^11^School of Veterinary and Life Science, Murdoch University, Perth, WA, Australia; ^12^Royal Veterinary College, London, United Kingdom

**Keywords:** transdisciplinary, integrated approaches to health, evaluation framework, one health, one health index, one health ratio

## Abstract

Challenges calling for integrated approaches to health, such as the One Health (OH) approach, typically arise from the intertwined spheres of humans, animals, and ecosystems constituting their environment. Initiatives addressing such wicked problems commonly consist of complex structures and dynamics. As a result of the EU COST Action (TD 1404) “Network for Evaluation of One Health” (NEOH), we propose an evaluation framework anchored in systems theory to address the intrinsic complexity of OH initiatives and regard them as subsystems of the context within which they operate. Typically, they intend to influence a system with a view to improve human, animal, and environmental health. The NEOH evaluation framework consists of four overarching elements, namely: (1) the definition of the initiative and its context, (2) the description of the theory of change with an assessment of expected and unexpected outcomes, (3) the process evaluation of operational and supporting infrastructures (the “OH-ness”), and (4) an assessment of the association(s) between the process evaluation and the outcomes produced. It relies on a mixed methods approach by combining a descriptive and qualitative assessment with a semi-quantitative scoring for the evaluation of the degree and structural balance of “OH-ness” (summarised in an OH-index and OH-ratio, respectively) and conventional metrics for different outcomes in a multi-criteria-decision-analysis. Here, we focus on the methodology for Elements (1) and (3) including ready-to-use Microsoft Excel spreadsheets for the assessment of the “OH-ness”. We also provide an overview of Element (2), and refer to the NEOH handbook for further details, also regarding Element (4) (http://neoh.onehealthglobal.net). The presented approach helps researchers, practitioners, and evaluators to conceptualise and conduct evaluations of integrated approaches to health and facilitates comparison and learning across different OH activities thereby facilitating decisions on resource allocation. The application of the framework has been described in eight case studies in the same Frontiers research topic and provides first data on OH-index and OH-ratio, which is an important step towards their validation and the creation of a dataset for future benchmarking, and to demonstrate under which circumstances OH initiatives provide added value compared to disciplinary or conventional health initiatives.

## Introduction

Many current health challenges, such as spread of zoonotic infectious diseases, environmental pollutants, antimicrobial resistance, climate or market-driven food system changes with consequences on food and feed supplies, malnutrition including obesity and many more arise from the intertwined spheres of humans, animals, and the ecosystems constituting their environment (1, 2). They are recognised to be wicked problems and need to be tackled using integrated approaches to health (3–5). Here, we consider integration as inter-^T1^ or transdisciplinary^T1^ (annotated terms are explained in detail in Table 1) approaches. Such approaches consider the needs, values, and opinions of multiple disciplines and sectors. They also bring together the scientific and non-scientific communities, influencing, or influenced by, the challenge and their combined know-how and resources (6–8). Due to the existing, historically contingent, separation of sectors and disciplines, developing integrated approaches is difficult, and the realisation of benefits can be delayed. There is a need to provide evidence on the added value of these integrated and transdisciplinary approaches to governments, researchers, funding bodies, and stakeholders (9–11).

**Table 1:** Glossary of terms and abbreviations used in this manuscript.

Term	Abbreviation	Explanation	Reference
Multi-disciplinary	MD	The multi-disciplinary approach is typically understood as the sequential or additive combination of ideas or methods	(8), or http://www.arj.no/2012/03/12/disciplinarities-2/

Interdisciplinary	ID	The interdisciplinary approach involves the integration of perspectives, concepts, theories, and methods to address a common challenge	(8, 12), or http://www.arj.no/2012/03/12/disciplinarities-2/

Transdisciplinary	TD	The transdisciplinary approach entails not only the integration of approaches, but also the creation of fundamentally new conceptual frameworks, hypotheses, and research strategies that synthesize diverse approaches and ultimately extend beyond them to transcend pre-existing disciplinary boundaries. The term transdisciplinarity refers to scholarship that transgresses the boundaries between academia and communities outside academia. By doing so, OH enables inputs and scoping across scientific and non-scientific stakeholder communities and facilitates a systemic way of addressing a challenge	(8, 12)

Sector		A sector is an area of activity aimed at benefits to society, characterised by common processes and institutions. Examples include agriculture, health, transportation, education, and environment. Sub-sectors would be units within the sector; for example, in agriculture these could be livestock, crops, agro-forestry, fishing, and aquaculture	

System, social-ecological system	SES	A system is a set of interacting, interrelated, or independent components that form a complex and unified whole (13). Human made systems are usually conceived to achieve a defined aim (14). However, this may not be the case for social-ecological systems (SES), which were defined as a hierarchy of subsystems and internal variables at multiple levels analogous to organisms composed of organs, organs of tissues, tissues of cells, etc. The core subsystems of an SES are resource systems, resource units, governance systems, and users (15)	(13–15)

Component		Systems are composed of a set of interacting or interdependent components that form a complex whole. Components may be tangible (e.g., humans, animals, forests, lakes) or intangible (e.g., cultural behaviours, values, norms, language expressions) and are linked by interactions	(13, 16)

Context		The system or SES within which the initiative is aiming to evoke change towards a health outcome	

Resource system		Resource systems are core subsystems of an SES such as forested areas, wildlife, water systems, national parks, etc. We extend the idea of Ostrom and consider social systems as resource systems too, e.g., health care system, local community, food chains, etc. They “provide” or host resource units such as trees, shrubs, susceptible persons, traders, food items, etc. which contribute to the system	(15)

Resource units		Resource units are product or component of the resource system and represent a link of the resource system to other components. In contrast to Ostrom, we do not differentiate between users and resource units, because users may represent a resource from, e.g., a disease perspective	(15)

Governance system		Governance systems are a further core subsystem of a social-ecological system and represent the system that is managing specific resource systems.	(15)

Stakeholder		Stakeholder is “*any individual, group or organisation who may affect, be affected by, or perceive themselves to be affected by a decision or activity*”	(17)

Actor		Actors are a subgroup of stakeholders such as “*any individual, group or organisation who acts, or takes part*” in the context of the OH initiative	(17)

One Health	OH	OH emphasises the commonalities of human, animal, plant, and environmental health. In this perspective, it can be regarded as an “umbrella” term that captures integrative approaches to health across these highly interlinked components	(7)

One Health initiative	OH initiative	Any initiative, such as research projects, developmental programmes, policy, etc. that relies on the concept of OH as described above. In a generic way, an OH initiative aims at generating change in a SES (context) towards improved health of humans, animals, and/or ecosystems. We do *not* refer to the *pro bono* Kahn–Kaplan–Monath–Woodall–Conti “One Health Initiative” at http://www.onehealthinitiative.com	(7)

Network for evaluation of One Health	NEOH	A network funded by the European Cooperation in Science and Technology (TD1404) with the aim to enable future quantitative evaluations of OH activities and to further the evidence base by developing and applying a science-based evaluation protocol in a community of experts	http://neoh.onehealthglobal.net

Evaluation design		A plan for conducting an evaluation	

Scale		Identical to level. Systems are organised in hierarchical order. This hierarchy implies that different levels of the hierarchy can be in the focus of attention. As an example in the hierarchy of life, one can look at individuals, populations, communities, or ecosystems, i.e., different scales of the same quality (life)	(18)

Level		Used as synonym to scale	

Dimension		Systems are organised in hierarchical order. Hierarchies depend on a fundamental quality that defines this order. Examples for dimensions are life with its different organisational levels; within the semantic space (dimension) expands the hierarchy of meanings of words; within the dimension of faith various beliefs are organised within larger clusters, but also governance, time, geographical space, and many more are dimensions	(18)

Space		Here used as synonym to dimension	

Theory of change	TOC	The TOC explains all the different pathways that might lead to the desired effect of an initiative. It not only shows the outputs, outcomes, and impact of an initiative, but also requires outlining (and explaining) the causal linkages. Each effect is shown in a logical relationship to all the others	(19) and http://evaluation.lshtm.ac.uk/process-evaluation/#toc

Logic model		Logic models graphically illustrate the components (inputs, activities, outputs, outcomes, impacts) of a programme in a structured, logical, and sequential way	http://www.theoryofchange.org/wp-content/uploads/toco_library/pdf/TOCs_and_Logic_Models_forAEA.pdf

Impact		Positive and negative, primary, and secondary long-term effects produced by a development intervention, directly or indirectly, intended or unintended	(20)

Output		The products, capital goods, and services which result from an OH initiative; may also include changes resulting from the intervention which are relevant to the achievement of outcomes	(20)

Outcome		The likely or achieved short-term and medium-term effects of an OH initiative’s outputs	(20)

Outcome mapping		An approach used for planning and assessing programmes that focus on change and social transformation. It provides a set of tools to design and gather information on the outcomes, defined as behavioural changes, of the change process	https://www.outcomemapping.ca/

For One Health (OH), as a typical integrated approach to health, the COST Action TD1404 “Network for Evaluation of One Health”^1^ (NEOH) was initiated to develop a science-based evaluation framework and apply it to a set of case studies (21). The NEOH framework uses a systems approach and regards the context of an OH initiative as the system within which it operates, and the initiative itself as a subsystem, which has a potential to affect the system to a smaller or larger degree. Drivers, operations, supporting infrastructure, and outcomes were identified as fundamental characteristics of any OH initiative (7). The NEOH evaluation framework relates the aspects of operations (i.e., OH thinking, OH planning, and OH working) and supporting infrastructure (i.e., systemic organisation, learning, and sharing) summarised as OH process characteristics (“OH-ness”), to changes and outcomes evoked by a specific initiative. This is an important step towards identifying added value arising from integration across disciplines and sectors (i.e., transdisciplinarity).

## Proposed Evaluation Framework

### Overview

**Figure 1:**
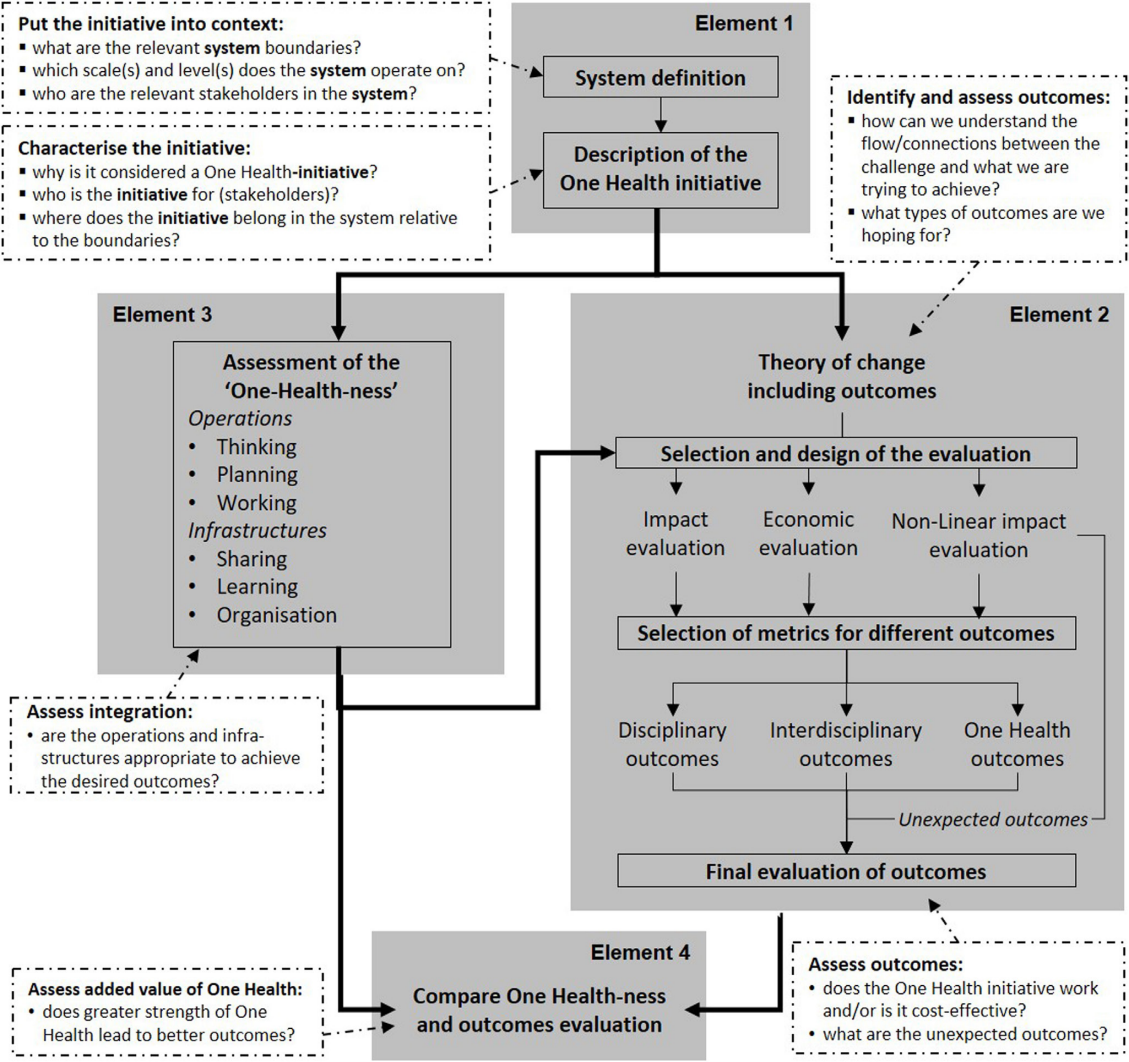
Flow chart of Elements to be considered during a One Health (OH) evaluation (in grey) with their purpose and the associated questions to be answered (white boxes). In Element 1, the initiative and its context are described to inform Elements 2 and 3. Element 2 relies on a theory of change to identify expected outcomes and collects unexpected outcomes through non-linear impact assessment. In Element 3, the implementation of operations and infrastructure contributing to the OH initiative is assessed. The two assessments are compared in Element 4.

Figure 1 provides an overview of the NEOH evaluation framework. There are four overarching Elements (grey boxes) in the evaluation process, namely:

**Element 1**: defining and describing the OH initiative and its context (i.e., the system, its boundaries, and the OH initiative as a subsystem), providing information for the further Elements;
**Element 2**: assessing expected outcomes based on the theory of change (TOC) of the initiative, and collecting unexpected outcomes emerging in the context of the initiative;
**Element 3**: assessing the “OH-ness”, i.e., the implementation of operations and infrastructure contributing to the OH initiative; and
**Element 4**: comparing the degree of “OH-ness” and the outcomes produced.

The framework relies on a mixed methods approach that combines a descriptive and qualitative assessment with a semi-quantitative evaluation (scoring) for the evaluation of the “OH-ness” with an OH-index, while including conventional metrics for outcomes in a multi-criteria-decision-analysis.

The framework can be used for either external or self-evaluation. It is recommended that the evaluator is comfortable with systems thinking (14, 22) to approach the complex structures and dynamics of OH initiatives and their context. Data and information can be gathered from actors^T1^ and stakeholders^T1^ using methods such as open or semi-structured interviews, focus group discussions, or other qualitative data collection approaches. These can stem from resources used or produced by the initiative (23), and related (external) primary or secondary datasets.

In the present manuscript, we describe a concept for the process of generating evaluation data (Elements 1–3), while Element 4 is analytical and is described in the evaluation handbook of the NEOH (for details see text footnote 1). The text is conceived as a set of short theoretical and methodological syntheses for each of these Elements. For their implementation, we present an exemplified application of Element 1 (definition of the initiative and its context) with a description and an illustration; an overview of categories of outcomes to consider in Element 2 (TOC and assessment of outcomes); and a short description of a consolidated file with six evaluation protocols (Table S1 in Supplementary Material) including OH-index calculations for Element 3 (assessment of OH-ness). For examples that apply the method presented here, the readers can refer to the case studies included in this Frontiers research topic on “Concepts and experiences in framing, integration and evaluation of OH and EcoHealth”.^2^ Paternoster et al. evaluated integrated surveillance of West-Nile virus (24), Radeski et al. applied the framework to an animal welfare centre (25), Léger and co-workers evaluated a research project on antimicrobial resistance involving four faculties, the industry, and health authorities,^3^ Buttigieg et al. compared control strategies for Brucellosis in Serbia and Malta,^4^ Muñoz-Prieto et al. assessed a study on factors affecting obesity in dogs and dog-owners,^5^ Laing et al. evaluated a project mitigating the effects of the unexpected domestic re-use of containers employed for organophosphates in a tick control programme (26), Fonseca et al. applied the framework to evaluate a cross-sectoral observatory of taeniasis and cysticercosis,^6^ and finally Hanin et al. evaluated an international and inter-sectoral centre for infectious disease surveillance (27).

### Definition of the Initiative and Its Context

Before designing an evaluation, the evaluation question(s) must be clearly stated. To answer these questions and to select an adequate evaluation design^T1^, it is then important to gain a principle understanding and overview of the activities to be evaluated (28). The framework presented here uses a systems approach and regards the context^T1^ of an OH initiative^T1^ as the system^T1^ within which it operates, and the initiative itself as a subsystem conceived to induce change in this context. Systems have been defined in many different disciplines and frameworks [e.g., Ref. (14, 21–24)]. A fundamental feature is that systems are composed of a set of interacting or interdependent components^T1^ that form a complex whole (13). This implies a hierarchical organisation and a concept of levels^T1^ or scales^T1^ within different dimensions^T1^ (18). Although the term “level” is used ambiguously in science, the concept used here is that of “grades of being ordered,” which captures what biologists and social scientists refer to as “levels of organisation” (29). Three such grades or levels can be identified at which OH outcomes are usually measured: individual level of health, population level of health, and ecosystem level of health (30). Systems can be considered as a network of components^T1^, which can be tangible (e.g., humans, animals, forests, and lakes) or intangible (e.g., cultural behaviours, values, norms, and language expressions) and which are linked by interactions (13, 16). The system’s components depend on the perspective and determine its boundaries, which are important for evaluation (23). While the perspectives of stakeholders (and thus system boundaries) may differ, the stakeholders may become agents of change or part of a pathway towards successful solutions (24, 26, 28). OH initiatives might create additional opportunities to produce relevant—expected as well as unexpected—outcomes by including stakeholders and system boundaries explicitly (Figure 1).

Element 1 of the evaluation framework (Figure 1) consists of a general overview (see the section “The General Overview”), a visual representation and a textual description of the system in which the initiative operates (see the section “Visual Representation and Textual Description of the Context”), and an analogous illustration and description of the initiative within this context (see the section “Illustration and Description of the OH Initiative within the Context”). They do not need to be developed in sequence, but may evolve iteratively, and may be developed by a group of evaluators or by the stakeholders of the initiative, or by the two groups in collaboration.

#### The General Overview

For the general overview, the evaluator should put together a concise description of the background, objectives, key features, and rationale of the OH initiative under evaluation so that the user is aware of the important characteristics that can affect the evaluation.

#### Visual Representation and Textual Description of the Context

Here, the focus is specifically on the system targeted by the OH initiative; in other words, the wider context within which the initiative operates. We will describe the initiative itself later. For the visual representation of the system, we propose a combination of the socio-ecological system framework by Ostrom and a causal loop diagram (13, 15).

**Figure 2:**
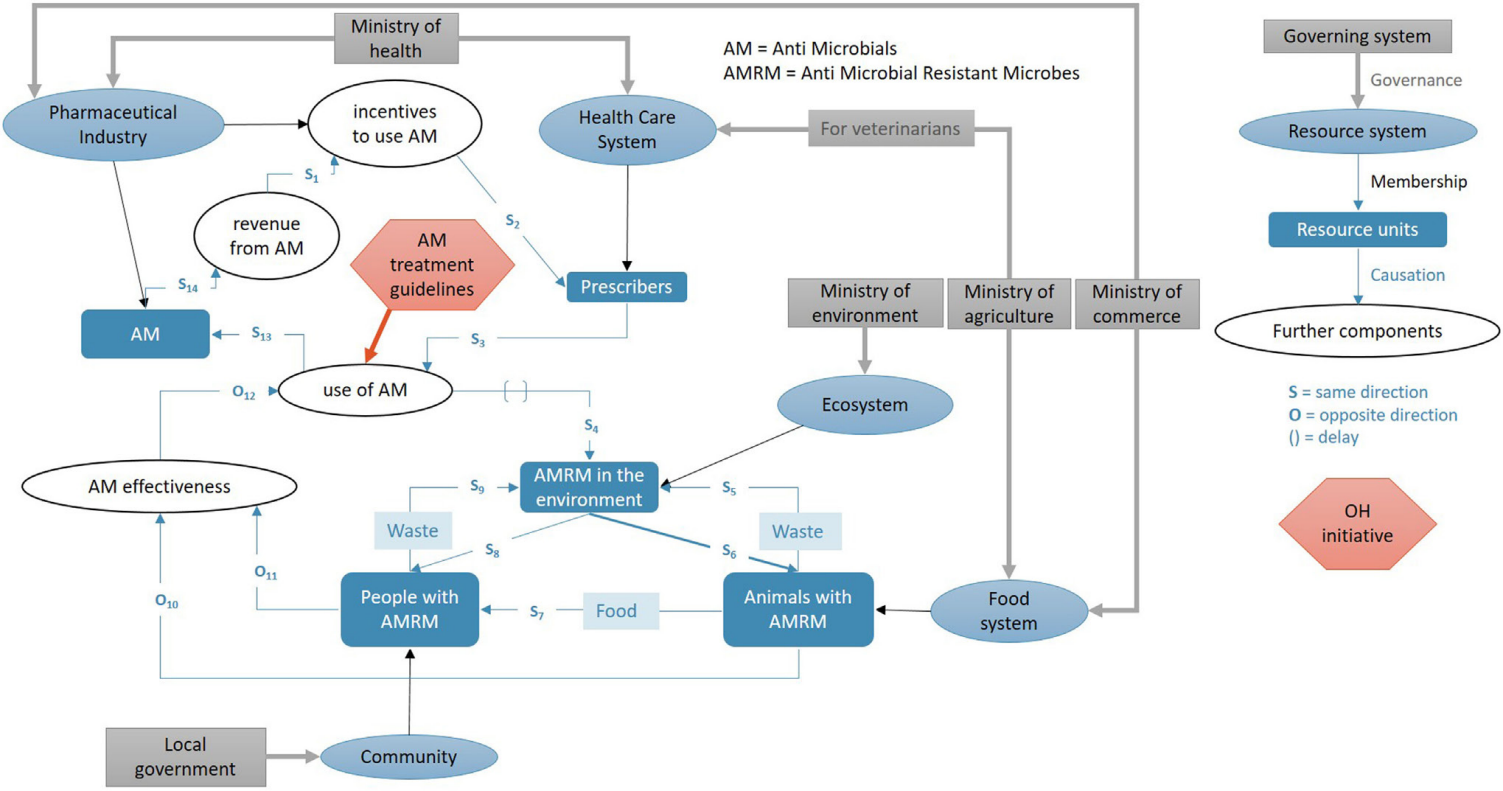
Example for visual representation of an initiative in its context exemplified by occurrence of antimicrobial resistance within a given system: resource systems (blue ovals), resource units (dark blue boxes), and governance systems (grey boxes) within which an initiative operates. Furthermore, tangible and intangible components (white ovals) are included. Relationships (arrows) are classified as governance (grey), membership (black), and causal interactions (blue) with explanatory text (light blue boxes). Letters designate changes of two components in the same (S) or opposite (O) direction, respectively. The red hexagon represents the initiative with arrows where it impacts the system.

To capture the socio-ecological system, three core subsystems are plotted first (Figure 2): the *resource systems^T1^* (blue ovals), the *resource units^T1^* they provide (dark blue boxes), and the *governing systems^T1^* (grey boxes). In the next step, further tangible and intangible components relevant to the system (white ovals, e.g., use of antibiotics, effectiveness of antimicrobials) are added. For legibility of the graph, it is recommended to use nouns that fit into phrases such as “the level of…,” to avoid verbs and to use neutral terms, e.g., “use of antimicrobials” rather than “increase of antimicrobial use.” Finally, *relationships* are added as arrows, namely governance relations (grey), membership relations (black), and causal relations (blue). For causal relations, it is useful to note the relation using S for “same direction change” and O for “opposite direction change,” in order to identify later reinforcing and balancing loops. Subscripts and explanatory text as well as annotations of time delays can be convenient for later reference.

Visual representation is powerful, but lacks any dimension beyond the plane and therefore hinders the depiction of overlapping subsystems or nested hierarchies. Hence, to explore further the system in which the OH initiative operates, it is recommended to include a textual description. It is guided by three questions formulated by Williams (28): (A) to understand interrelationships: what is the reality we are dealing with? (B) to engage with perspectives: how do we understand/how do we see that reality? (C) to reflect on boundaries: how do we decide to do what needs to be done? (28). In Table 2, we adapted the tabular system description by Boriani et al. (31) for a broader application. It allows capturing the aim of the system, the stakeholders and actors and their interactions, the system dimensions with corresponding boundaries, and the system evolution.

**Table 2:** An overview of how to describe the system at which the One Health (OH) initiative is targeted, i.e., the context of the initiative.

Aspect	Description	Secondary questions	Evolution
Aims	What is the context of the OH initiative—why does this system exist? What does it produce? For social-ecological systems that have no explicit aim, what are indicators that the system is intact/healthy?	Perspectives What does the system declare to do? Are there different declarations? What do the actors and stakeholders perceive the system does and how do those perceptions differ? (For social-ecological systems: how do the actors and stakeholders perceive/evaluate that the system is intact/operational?) Are there measurable outcomes/indicators of the system? How do the declared, perceived and measured aims/outcomes relate?	Do the various aims/indicators change as the system evolves with time?

Actors	Who are the actors? Who acts within the system?	Relationships How do they affect the other actors/stakeholders and the aim of the system? How are they affected by the other actors/stakeholders and the aim/indicators of the system? How are the relationships distributed/arranged? Which are the most important links? What are the processes between the related components? How can the links be characterised (slow/fast, strong/weak)?	Do the actors change their activity and behaviours as the system evolves (new trade-offs)? Does the system have secondary effects on the actors?

Stakeholders	Who are the stakeholders? Who is affected by the system?	Relationships How are they affected by the actors and the dynamics of the system? How are the relationships distributed/arranged? Which are the most important links? What is the nature of the processes between the related components? How can the links be characterised (slow/fast, strong/weak)?	Does the system have secondary effects on the stakeholders?

Geographical dimension	Which geographical space does the system occupy and where is it situated (surface concerned, climate, and location)?	Boundaries How is the system delimited in geographical area? How do these boundaries affect the system aims/indicators and dynamics?	Does the system have secondary effects in geographical space within the boundaries? Does the system produce “externalities” in geographical space?

Temporal dimension	Which is the most important time scale in which events are happening in the system (e.g., minutes, months, and years)? Are there other important time scales?	Boundaries How is the system delimited in time? Is it infinite, terminated, transient? How does this time limit affect the system aims/indicators?	Does the system affect the frequency of events or its own time limit? Does the system produce “externalities” in time (accelerating or slowing down external systems)?

Governance/institutional dimension	Which governance entities/levels are involved (shire, agglomeration, state, nation, or international space)? What institutional structures (companies, corporations, and organisations) play a role?	Boundaries How is the system delimited in the governance/institutional dimension? How do these boundaries affect the system aims/indicators?	Does the system have secondary effects in the governance/institutional dimension within the boundaries? Does the system produce “externalities” in the governance/institutional dimension?

Further dimensions	How does the system extend within this dimension and how many levels of this dimension are part of the system?	Boundaries How are these dimensions delimited? How do these boundaries affect the system aims/indicators?	Does the system have secondary effects in these dimensions within the boundaries? Does the system produce “externalities” in these dimensions?

*The aim and/or indicators of the system* are not to be confused with the aim of the initiative and should answer the question “why does the system exist?” or “what does it produce?” e.g., the result of a food chain may be to “produce Salami.” A social-ecological system may not have an explicit aim, but it can be characterised by indicators that allow describing selected attributes, such as resilience, productivity, or health. In this evaluation framework, we differentiate among the declared aim by the system and the observed, enacted, and the perceived aims. The declared aim of a veterinary practice may be to provide animal health services. However, this will be enacted within a socio-economic context, which may result in therapeutic choices that prioritize practice income over animal welfare. These actions may be observed by a subset of clients, while others do not notice them. Each stakeholder may have a different perception of the declared aim and again, each of them can have a different way to interpret how the system is performing in relation to its aim (13). In socio-ecological systems, the perceptions differ mainly in regard to the way one verifies whether the system is healthy and/or intact. This is important as it explains the motivational background and sets of values of the concerned stakeholders. Indicators specific for the system aim should be identified in a participatory process and compared with indicators used by different stakeholders to assess their perceived aim(s), thereby shedding light on discrepancies and ways of resolving them.

Following the interactive terminology for Europe (17), we define *stakeholders^T1^* as “*any individual, group, or organisation who may affect, be affected by, or perceive themselves to be affected by a decision or activity*,” while actors^T1^ are a subgroup of stakeholders such as “*any individual, group, or organisation who acts, or takes part”* in system activities. To gain clarity about roles of stakeholders, we recommend referring to the visual representation of the system exemplified in Figure 2 and probe for “who is involved in the system as an *actor* and who is merely *affected*?” For example, the pharmaceutical industry produces a certain compound, people can decide whether to take that compound or not, while animals are affected by a certain preparation distributed to them by an actor in the system (e.g., veterinarian or owner). An overview of relevant actors and stakeholders allows delimiting further the system under evaluation. Stakeholders could be actors at the same time, and in these situations, it should be differentiated in what capacity a group represents a stakeholder or actor, respectively.

In order to understand the context of the OH initiative, it is important to understand how the components of the system are arranged or interact (28). There are four aspects of relationships that should be considered and described: (a) the structure or arrangement of the links between the components (topology); (b) the nature of the processes between the components (e.g., information flow, transfer of goods, etc.); (c) the characteristics of the links (slow/fast, strong/weak, antagonistic/synergistic, etc.); and (d) identifying the links that are most important in the system.

*Dimensions*^T1^ are defined as spaces in which levels of organisation according to Bunge occur (29). In other words, entities within a dimension feature the same quality (e.g., metric) but to a different degree. Examples include geographical space, time, governance/institutional, economic, linguistic, faith, and value dimensions. Within these dimensions, we consider scales^T1^ or levels^T1^ of analysis, e.g., cell—organism—population in the dimension of life (18). These levels are important, because they will determine the relation between the resolution of the analysis and the resolution of observations and what can be measured or evaluated in the system in a particular dimension. Due to their importance, *geographical, temporal*, and *governance/institutional dimensions* are included in Table 2. Particularly time is related to the scale in other dimensions, i.e., the larger the system the larger its characteristic time, which is the time at which the average change occurs (e.g., cells react within milliseconds, individuals within minutes to hours, ecosystems is within years or decades, the same applies to the adaptability of laws at different scales or the frequency that vocabulary is used in a language) (18). Together with geographical space, time is a particularly important dimension, because it will characterise if the system is evolving over seconds, hours, days, years, decades, or even longer. It can be considered in the past, present, or future, and opportunities to affect the system are highly dependent on time due to the system disposition (the same intervention may have different effects when applied at different times). Furthermore, causes and effects may occur in different time scales, where short actions may result in effects with a time lag of years. The governance/institutional dimension will determine which organisational levels (ranging from international governance mechanisms to household structures) are represented and addressed in an initiative. Considering scales is important, because initiatives may aim to change systems at different levels than where the necessary governance could be influenced and consequently, well intended initiatives may remain ineffective if they do not address all appropriate levels.

Further dimensions are the *Dimension of Life* (or Biology) comprising nested living entities from cells to biosphere with levels such as “cell,” “organ,” and “individual,” the *Economic Dimension* defined by rules and institutions involved in decisions on production, trade, and exchange of goods and services, the *Linguistic Dimension* delimited by languages and dialects used, the *Faith/Value Dimension*, which represents the values and beliefs underlying the system. Other dimensions may also be relevant to the system, such as communication, transportation, legal frame, sociocultural dimensions, and many others.

The primary importance of a systems approach to evaluation implies less the idea of being comprehensive, but rather being “thoughtful, smart, and aware about what you are leaving out” (28). The evaluator(s) will need to be transparent about the consequences of choices and declare their relation to the initiative, the system, and the evaluation *per se*. Although the dynamics and boundaries and stakeholders of a system are clear, they will be constrained by physical limits (e.g., a mountain range, river), social limits (e.g., country, community), regulations (e.g., quotas, prohibitions), and/or other norms (e.g., social norms, religious norms) that are either imposed by the systems nature or selected by the evaluators (23). Many restricting factors will lie in one of the system dimensions identified earlier. For example, a food system can be limited due to production regulations (e.g., the previous milk quotas system in Europe), food hygiene standards (e.g., restrictions on raw milk consumption), or cultural practices (e.g., no pork consumption in certain faith groups). The system boundaries characterise the interaction between the context of the initiative with the broader world in which it is imbedded, and determine how this affects the aim of the system (23). Finally, dimensions can also interact and may even be so closely correlated that it may not be useful to differentiate them (e.g., when religious beliefs are prescribed by the law).

The *evolution of a system* can be regarded as interaction of time with other dimensions in terms of iterations and pathways along those dimensions and time. Apart from the aim of the system, the interactions in the system may produce secondary effects within the system and “externalities” beyond the boundaries as it evolves. Highly self-organising systems may even change their (aim) dynamics and boundaries as time goes by.

#### Illustration and Description of the OH Initiative within the Context

In a next step, the OH initiative can be added to the visual representation of the context to illustrate its effects on various components^T1^ and their interactions. If an affected component is missing, it is added and the system graph corrected accordingly. In the example in Figure 2, we have included a hypothetical OH initiative that involves new antimicrobial treatment guidelines for veterinarians and general practitioners (prescribers) that are assumed to impact directly on the amount and distributions of types of antimicrobials used in the system.

The user should now have a clear understanding of the system in which the OH initiative is situated. Next, the initiative itself is described using the template in Table 2 in analogy, namely as a nested subsystem of the context, which it aims to change. Many elements may be congruent, but the boundaries of the initiative will inevitably be smaller and there will be fewer actors, stakeholders, and more limitations than in the description of the system. Care should be taken, as actors and stakeholders and their particular roles, may not be identical in the initiative and in the wider system. The initiative may be likely to consider fewer dimensions compared with the system, but it is important to identify how it will influence the context and what the limitation of the actions are. A key question in this description is: how is OH conceptualised by the various participants and is there a common understanding?

### TOC and Assessment of Outcomes

Element 2 involves elaborating the TOC^T1^, which helps to explain how an initiative is intended to produce the desired (or expected) outcomes. It is an important step to define the evaluation question and to choose the evaluation methods and metrics. It entails generating hypothesis about the causal mechanisms by which the components and activities of the initiative produce outcomes by asking pertinent questions about: (A) why people expect the initiative to bring about the change(s) and the outcome(s) they seek, (B) to question their assumptions about how the change process will unfold, and (C) to be clear about how they are selecting outcomes for the evaluation. Identifying and developing a theoretical understanding of the likely process of change is critical when evaluating complex initiatives (32). Measuring (or assessing) change in multiple outcomes, facilitates the evaluation of whether the OH initiative works as intended and whether it is cost-effective. In addition, unexpected outcomes may arise from an OH initiative. A good description and understanding of the system and OH initiative in Element 1 facilitates the identification of interactions and dynamics that may lead to unexpected and indirect outcomes not specified by the TOC. This framework standardises the evaluation through a systematic approach based on the TOC, while explicitly remaining open for potentially emerging systemic effects through the non-linear impact evaluation (Figure 1).

#### Description of the TOC

**Figure 3:**
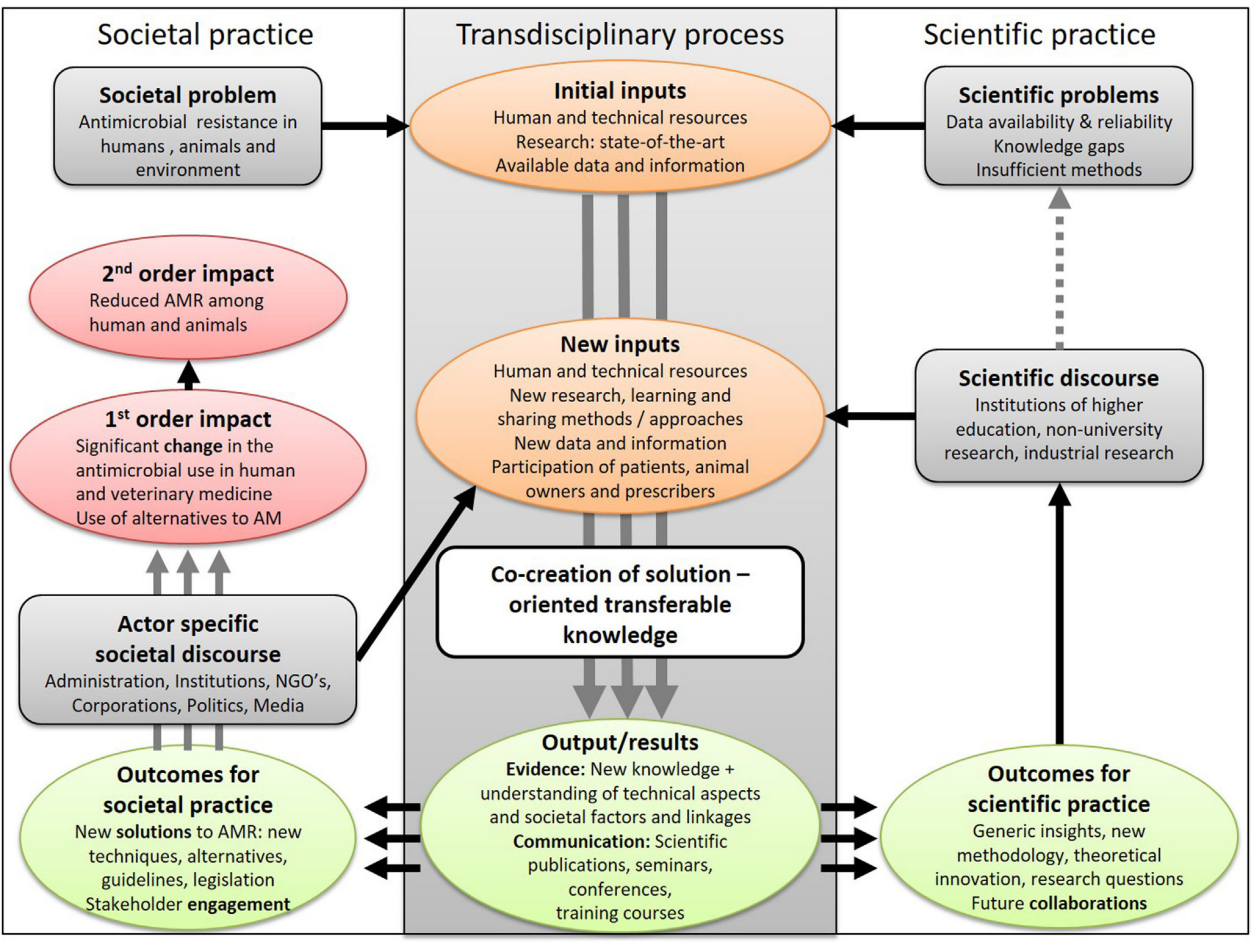
The change pathway for a fictive One Health research initiative aiming to mitigate the development of antimicrobial resistance in a transdisciplinary process. It illustrates the inputs from science and society to co-produce outputs that are taken up by society and the scientific community and disseminated through a specific discourse before resulting in first- and second-order impacts and scientific progress. On the way to impact(s) several iterations with new inputs and outputs of the transdisciplinary process may be needed.

Essentially, the TOC presents a roadmap with all building blocks required to bring about a desired (long-term) goal and hence spells out the logic behind the initiative. The presentation of the TOC can be assisted by a graphical presentation (e.g., Figure 3), or its description can refer back to the illustration of the system used in Element 1.

The impact^T1^ is defined as the long-term effects (or goals) to be induced by an OH initiative. It is a change that continues to exist after the end of the initiative, and can be direct (first order) or indirect (second order) impacts. Outcomes^T1^ are changes (e.g., improvement, learning) resulting from the initiative that can be considered to be stepping stones for progress towards the longer-term goals. In a transdisciplinary process, the outcomes are situated in societal and scientific practice and can be of multiple natures (e.g., technical, economic, social, sanitary, and political) (33). Outputs^T1^ are products, goods, and services, which result from the transdisciplinary process of an OH initiative and are necessary for the achievement of outcomes. For illustration, we use an example from a fictive research project aiming to produce new knowledge and methods to combat the development of antimicrobial resistance (Figure 3): OH research outputs (new data and knowledge) result in new treatment guidelines (outcome for societal practice) leading to new regulations restricting (and hence lowering) the use of specific antimicrobials in farmed animals (first-order impact of political nature), which then may reduce the development of antimicrobial resistance in farmed animals and the associated transmission to people (second-order societal impact). The impacts can be realised at different political levels (e.g., individual, institutional, regional, national, and international) and can consist of different types of effects (positive or negative; direct or indirect). Outcomes for societal and scientific practice (e.g., an improved integrated surveillance programme for antimicrobial resistance or a new simulation model, respectively) are disseminated, adapted, and applied by other actors to result in societal impact or scientific progress. Between the initial problem formulation and the expected impact(s), new inputs might be required as a result of intermediary outcomes and will feed a further iteration of knowledge co-production. An example could be new research collaborations as the outcome of an OH initiative, which may lead to new knowledge or tools for improved control of infectious diseases in a second initiative. The sequence of inputs (i.e., resources needed to perform the actions), outputs, outcomes, and impact can be graphically represented by a change pathway also known as an impact pathway (34) or a logical framework^T1^ or logic model^T1^, which presents the flows in a “logical,” sequential way (19). Importantly, the classification into outputs, outcomes, and impacts depend on the perspective that is taken for the evaluation and may differ among stakeholders (35). It is therefore important to elaborate the TOC in collaboration with the entity contracting the evaluation.

A methodology related to the TOC is outcome mapping^T1^, which can be used for planning and assessing (development) activities focusing on change and social transformation. It places people and learning at the centre of development and conceptualises unanticipated changes as potential for progress and innovation. Consequently, it can be a useful tool to use for OH initiatives, either in combination with TOC or on its own if it fulfills key assumptions of dependence on human behaviour, limits to the influence of interventions, active contribution of people to their well-being, co-existence of differing yet valid perspectives, and resilience dependent on interrelationships (36).

#### Expected Outcomes and Impacts

The description and definition of outcomes and impacts are dependent on the problem the OH initiative is addressing and the associated boundaries of the system, objective, rationale, and consequently the resulting TOC. Given the diversity of OH initiatives, there is no single outcome that summarises OH endeavours, but rather a wide range of different outcomes (37–39). However, at the longer-term impact level, there are commonalities OH endeavours appear to strive for (7). The outcomes and impacts to be measured need to be selected as best fit for the specific OH initiative and its TOC. Because of their nature, OH initiatives will commonly span different sectors and disciplines and therefore are likely to produce disciplinary, interdisciplinary, and OH outcomes and impacts. Evaluators consequently need to be aware of disciplinary paradigms, data, and approaches as well as methods of combining outcomes from different disciplines.

*Disciplinary outcomes* relate to outcomes that are measurable within a distinct discipline or sub-speciality within the natural or social sciences. Examples of disciplinary outcomes include health outcomes such as decreased levels of non-communicable or infectious diseases; nutrition outcomes such as reduced levels of undernutrition or obesity; economic outcomes such as increased productivity or savings in the health care system; social outcomes such as improved societal stability; and ecological outcomes such as slower rates of biodiversity reduction or improved water or air quality. Importantly, these outcomes can be achieved in disciplinary or sectoral approaches (e.g., promotion of a new anti-diabetes treatment or childhood vaccination in a national health service), but more often, they rely on collaborations across disciplines and sectors. Interdisciplinary activities by definition have an impact on multiple fields or disciplines and produce results that feed back into and enhance disciplinary or sectoral work. In these instances, the pathway to the outcome may be characterised by collaboration and contributions from different disciplines and sectors, but the outcomes may still be conceptualised (and consequently measured) at the level of a field or discipline. Combining these disciplinary outcomes in methods such as multi-criteria decision analysis gives a solid basis for an assessment of the achievements of the OH initiative. In *interdisciplinary outcomes*, individuals from different disciplines create new knowledge and understanding through sharing of ideas and bringing together different perspectives result in a product or measure, which explicitly reflect the shared responsibility among disciplines for outcomes (16, 22, 40). Consequently, interdisciplinary outcomes occur in the realm of at least two disciplines simultaneously, e.g., food security as an interdisciplinary outcome of successful alignment of multiple sectors (i.e., food availability, food access, and food utilisation), which contribute different skills and expertise (41). Other examples are the human development index, the environmental performance index, and the planetary boundaries, which combine a diversity of indicators into a single or a few measure(s). An improvement in the index cannot be achieved with a disciplinary approach, but needs activities in health (e.g., investment in health service capacity, public awareness campaigns), education (e.g., build infrastructure, attracted attract talented teachers, and provide incentives for school attendance), social protection (e.g., policies to reduce poverty and vulnerability of disadvantaged population groups), and economics (e.g., promotion of efficient labour markets, robust governance). Interdisciplinary outcomes are ideally measured in a common metric, i.e., they should rely on a consensus on how to assess and weigh the particular outcomes. Such metrics are even more policy relevant and effective if they are produced and measured in a transdisciplinary process, which transcends both horizontal boundaries between scientific disciplines, and vertical boundaries between science and other societal fields (private sector, public agencies, and civil society) (42). Like this, stakeholders share different perspectives and can therefore improve the contextualization of the problem and its potential solutions and targets (43).

*One Health outcomes* or impacts occur as result from a broader integration of activities in the system at stake. The main domains of OH outcomes are the three pillars of sustainability, i.e., society, environment, and economy. Typical examples are interspecies equity, health stewardship, human and animal welfare, efficiency, and effectiveness (7). Clear causal attribution to the OH initiative may be difficult, but a contribution of the OH initiative can be assessed.

Given the perspective chosen and the resource availability for the evaluation, the description of the TOC, and the selection of associated outcomes may be more or less comprehensive and complex. However, the evaluator should make sure to pay careful attention to the contributions from different disciplines and sectors, their integration and the resulting positive and negative effects.

#### Unexpected Outcomes and Impacts

By definition, unexpected outcomes and impacts cannot be planned or covered by a TOC, even though attempts are sometimes made to capture a wide range of eventualities. Throughout an OH initiative within its system, interactions among components and feedback loops frequently produce rapid, non-linear, and unanticipated changes (23, 44, 45). Typically, integrated approaches in complex systems generate unexpected added value, e.g., a new stakeholder organisation, but may also result in unexpected negative impacts, e.g., discrimination among stakeholders (23), which is why capturing unexpected outcomes constitutes an essential process of OH evaluation. Other examples would be emerging diseases due to new contact rates or closer contact between previously isolated populations, or due to new social behaviours in urbanised environments (46). If unexpected outcomes are not captured, evaluation fails in informing adaptive management that seeks to improve outcomes in complex dynamic environments (47). An expanding array of qualitative and quantitative methods for complexity-enabled monitoring, evaluation and learning is available for use in the fields of development and peacebuilding (48–50), many of which can be contextually adapted for OH projects and programmes.

### Assessment of OH-Ness

Aspects of implementation of initiatives (i.e., the structures, resources, and processes through which delivery is achieved, and the quantity and quality of what is delivered); mechanisms of impact (i.e., how activities, and participants’ interactions with them, trigger change); and context (i.e., how external factors influence the delivery and functioning of activities) are examined through process evaluation (51, 52). Process evaluations allow seeing how an initiative develops, its structures, environment, and associated activities like communications and marketing. An implicit characteristic of any OH initiative is its focus on sharing, exchanging, collaborating, learning (from each other), reflecting and generating change across disciplines, and sectors in an enabling environment (7). Consequently, this affects the delivery of an OH initiative (e.g., availability of training, learning about other fields, provision of resources), the mechanisms of impact (e.g., the responses of participants and their interactions with the initiative), and context factors (e.g., shaping of theories on how an initiative works). We refer to the sum of these characteristics as OH-ness composed of six aspects outlined below and hypothesise that they need to be an integral element of any (process) evaluation in OH. We collate scores and indices that have been suggested in a variety of contexts, adapt them to OH, and combine them in a OH-index (OHI) and OH-ratio (OHR) for a holistic appreciation. The six assessment tools have been standardised for use and are made available together with the calculation of the indices and automatic spider diagrams in an Excel workbook for download (Table S1 in Supplementary Material). Each assessment tool consists of a series of up to 17 questions to be answered and an associated scoring system with values between 0 and 1 as well as spider diagrams. The questions were developed by working group 1 of the NEOH and probe for the specificities of each aspect (outlined below) that can be captured in a semi-quantitative way. They are based on the concept of SMART goals (specific, measurable, achievable, relevant, and timely) and wherever appropriate, were adapted from existing evaluation tools. They were then circulated in the NEOH community and revised in several workshops throughout the Action. The scoring recommendations were determined so that scores close to one reflect a high degree of realisation of the different OH characteristics. Here, it must be emphasised that the authors do not presume that a high degree of implementation necessarily results in a high impact or effectiveness and underline that at this stage, the benchmark still needs to be established. Each question has the same weight, with exception of the learning assessment, where different levels of organisational learning are weighted according to their level of influence on institutional learning. Consequently, care was taken to balance the number of questions across all assessment tools to provide equal representation in the overall OHI. The underlying assumption is that each question contains equivalent information to describe the OH initiative. However, because there is no measurable gold standard for each of the questions, the questionnaire and primarily the OHI, and OHR are then assessed for their usefulness and representativeness using case studies as outlined in the overview and a meta-analysis of further published studies. Similar to Element 1, the assessment of the characteristics in this element should ideally be informed by a group of evaluators or (preferably) by relevant stakeholders identified in Element 1.

#### OH Thinking: System Thinking and Match between Context and Initiative

One Health as a systemic approach with corresponding methodology is of little worth if not based on a foundation of systems thinking (14). This tool assesses how an OH initiative conceptualises the system in which it operates and in how far it considers features specific to complex adaptive systems. The fundamental idea is that a complex initiative addresses multiple dimensions of the system in which it operates (see Element 1 above). The first set of questions (Table S1 in Supplementary Material) measure the number of dimensions and the scales within each to gain a semi-quantitative appreciation of the context and the embedded OH initiative. The following questions assess the match between the dimensions of the initiative and its context. Particular attention is given to the scales in different dimensions and whether the initiative reflects the reality of the context in which it operates. A third set of questions probes for concepts and thoughts typically contained in a systems approach (13, 53). To assess systems thinking in written documents, e.g., in a retrospective evaluation or in a proposal, we refer to a method based on statistical semantics proposed by Whitehead and Scherer (54).

#### OH Planning: Cross-Sectorial, Integrated Planning

One Health planning is essentially the unfolding of the OH thinking into operational features of the initiative that should facilitate OH working towards achieving the aims and objectives during as well as after the OH initiative. The planning of OH initiatives go beyond the type of planning that is required for disciplinary and interdisciplinary projects in which it might be easier to maintain control of what tasks, engagement, and resources are required. For instance, OH initiatives typically require human resources with competences in transdisciplinary working methods and excellent communication skills to bridge disciplines and sectors (8). It is important that the planning includes appropriate methods to engage all of the essential actors and stakeholders, who should be aiming to reach a common goal. Part of the planning evaluation is to assess whether the planned structure, location, and timing of the initiative support the OH outcomes aimed for. Due to the complex and trans-domain characteristics of OH challenges, another important aspect of OH initiatives is the ability to self-assess, learn, reflect, and adapt to new knowledge and changing conditions, constraints, and opportunities over time (55). Therefore, adaptability features prominently in the evaluation of the planning of OH initiatives. Finally, the planning evaluation helps assessing the tasks and resources allocated to each task employed to achieve the specified objectives of the initiative. The questions in Table S1 in Supplementary Material were developed to probe if the challenges of complex initiatives described here are addressed in the planning phase and if funding as well as organisational aspects are set up to accommodate adaptive behaviour by the participants. High scores are recommended for a strong support of adaptability and flexibility.

#### OH Working: Transdisciplinarity

Interdisciplinary collaboration brings together people with different skills and expertise to tackle complex problems, which often have a high-societal stake and require an understanding of the human behaviour (9, 56, 57). Appreciating potential contributions of multiple disciplines requires examining the limits imposed by a discipline, and rejecting or accepting different disciplinary theories based on their relevance and credibility in order to gain a new understanding about the defined challenge (12, 58). In the context of OH, interdisciplinarity^T1^ has developed towards a participatory approach in the form of transdisciplinarity^T1^ (57). Both inter- and transdisciplinarity rely on appropriate leadership and management to promote strategic dialogue and shared decision-making (40, 59), which in turn will foster a non-hierarchical relationship between the different disciplines and members within the team. It must also allow for self-reflection, flexibility, and recursiveness (40, 42, 57, 60), to be able to challenge and modify underlying assumptions and concepts and thereby enrich understanding. It must be emphasised that such transdisciplinary work demands a high level of commitment and collaboration of all participants to establish personal relationships founded within a climate of trust (9, 42, 59). The questions probing for transdisciplinarity (Table S1 in Supplementary Material) focus on disciplinary diversity, team building, and adaptability and were adapted based on the work cited above.

Further aspects of trans- and interdisciplinarity may be assessed, namely for (A) evaluating (academic) participants and (B) assessing scientific outputs of an OH initiative. However, because individuals may have different roles in an OH initiative, assessing their trans- and interdisciplinary capacity may not always be required or relevant. Also, printed scientific output may not be a primary objective of an OH initiative and occurs with some delay, thereby contributing more to the assessment of outputs than to the implementation *per se*:

(A) The transdisciplinarity of (academic) participants may be assessed based on the interdisciplinarity of publications [see method (B) below]; interdisciplinarity of teaching, other academic activity (e.g., teaching experience in other disciplines than the own, co-teaching with experts from other disciplines/sectors, etc.); previous experience with various non-academic communities (e.g., public debate, main stream media, sports and leisure organisations, politics, NGOs, volunteering, etc.); involvement in other disciplinary and interdisciplinary networks (e.g., social and natural science networks other than the own expertise, explicitly interdisciplinary initiatives, science policy, etc.);
(B) A framework to evaluate the interdisciplinarity of knowledge production based on citation network analysis can be found here: https://www.mcgill.ca/msr/msr-volume-4/evaluating-knowledge-production-systems. It must be emphasised that this only represents the written knowledge published in peer reviewed journals, which does not reflect the actual knowledge production occurring in the field.

#### Systemic Organisation: Adaptive and Shared Leadership

In many complex settings, change-oriented leadership has helped to overcome the fallacies of conventions, norms, and traditions (61, 62). Complex systems have leverage points where they can be influenced according to their potential to modify a systems behaviour (53). The use of these points by an OH initiative determines the dimension(s) and scales at which the initiative is effective. However, in order to be effective, the implementation of the initiative needs to be facilitated by corresponding leadership behaviour. Yukl classifies leadership into four meta-categories with specific objectives (62): for (A) task-oriented behaviour, the primary objective is to accomplish work in an efficient and reliable way. For (B) relations-oriented behaviour, the primary objective is to increase the quality of human resources and relations, which is sometimes called “human capital.” For (C) change-oriented behaviour, the primary objectives are to increase innovation, collective learning, and adaptation to the external environment. For (D) external leadership behaviour, the primary objectives are to acquire necessary information and resources, and to promote and defend the interests of the team or organisation. These leadership behaviours can be related to the leverage points in a system according to their objectives (Table 3).

**Table 3:** Ranked list of leverage points at which to intervene in complex systems, from least to most effective, according to Meadows (53), in relation to leadership behaviour according to Yukl (62).

Leverage point	Leadership behaviour
Constants, parameters, numbers (such as subsidies, taxes, and standards)	Task-oriented leadership: clarifying, planning, monitoring, and problem solving
The sizes of buffers and other stabilising stocks, relative to their flows	
The structure of material stocks and flows (such as transport networks, population age structures)	

The lengths of delays, relative to the rate of system change	Relation-oriented leadership: supporting, developing, recognising, and empowering
The strength of negative feedback loops, relative to the impacts they are trying to correct against	
The gain around driving positive feedback loops	
The structure of information flows (who does and does not have access to information)	
The rules of the system (such as incentives, punishments, and constraints)	

The power to add, change, evolve, or self-organise system structure	Change-oriented leadership: advocating change, envisioning change, encouraging innovation, and facilitating collective learning
The goals of the system	

The mindset or paradigm out of which the system—its goals, structure, rules, delays, parameters—arises	Change-oriented and external leadership: networking, external monitoring, and representing
The power to transcend paradigms	

Yukl emphasises that all leadership behaviours and particularly their flexible applications are relevant for effective leadership. The table simply illustrates that the lack of a particular leadership behaviour may hamper the implementation of a well-conceived OH initiative. The effectiveness of leadership behaviours also depends on the extent to which the leader is trusted by people to be influenced. Most types of leadership behaviours can be used in ethical or unethical ways. Moreover, a leader, who is not trusted because of unethical behaviour will have less influence. Values, namely honesty, altruism, compassion, fairness, courage, and humility may further catalyse effects of good leadership behaviour. In contrast, excessive institutional structure and organisation can nullify these effects (62). Rooke and Torbert identify further common personality traits of leaders that effectively manage wicked problems: they can challenge the prevailing view without provoking outrage or cynicism; they can act on the big and small picture at the same time, and change course if their chosen path turns out to be incorrect; and they lead with inquiry as well as advocacy, with engagement as well as command, operating all the while from a deeply held humility, and respect for others (63).

A further challenge for leading OH projects is that there may be less interest, commitment, and collaboration if one discipline dominates. Consequently, other disciplines may retract their activity and reinforce the disciplinary silo mentality. To ensure that disciplines are effectively engaged and involved in decision-making from the planning to the implementation stages of projects, shared/distributed leadership, and governance should be implemented involving all stakeholders (64, 65).

Consequently, the selection of questions for the systemic organisation of OH initiatives focuses on the structure of teams, as well as management, social, and leadership skills of key players and its implementation (Table S1 in Supplementary Material). The questions were taken from the leadership assessment tools and the published questionnaires on team work and transdisciplinarity described in Section “OH Working: Transdisciplinarity”. High scores were recommended for strong teams, change-oriented leadership skills, clear competences, goals, and criteria of success.

#### Learning Infrastructure

Learning is a change in cognition, potential behaviour or actual behaviour through better knowledge, and understanding (66, 67). Organisations, such as OH initiatives, learn when they “encode inferences from history into routines that guide behaviour” (68). This is achieved when discoveries, evaluations, and insights by individuals are successfully embedded in the organisation’s mental models or cognitive systems and memories (69). This requires that organisational learning takes into account the learning that takes place at the individual, group, and organisational levels (70) and the interplay between them (69). The three levels of learning work together and influence each other and are thus not clearly distinct and mutually exclusive (71). Nevertheless, each level of learning has its characteristics for evaluation.

Individuals can engage in single-loop or double-loop learning. Single-loop learning happens when the output is corrected or existing competences, procedures, technologies, and paradigms are improved, without necessarily examining or challenging the underlying beliefs and assumptions. In contrast, double-loop learning involves seeing beyond the situation and questioning operating norms. It results in modification of the organisation’s underlying norms, policies, and objectives.

Individual learning is not a sufficient condition for organisational learning (72). Teams enable the interplay between individual and organisational learning, because they can better share the knowledge (72–74) and include more people in the learning process. As a result, team members share awareness of each individual member’s expertise, knowledge, and skills, and build a transactive memory system (8). Thus, the evaluation should examine the knowledge shared through teams, to what extent it is shared and how it is shared. The conclusion should show whether the teams provide the appropriate interplay between the individual and the OH initiative. Without supporting the development of a transactive memory system within and across teams, the initiative may have individuals who learn, but it cannot engage in organisational learning (75). It is important to assess how knowledge is gathered, stored, and distributed within an OH initiative (76), and if and how it provides working environments, technology, rewards, systems, structures, and policies that will support learning (73).

Finally, the context in which the OH initiative is located has influence on the organisational learning (77). The context can be divided into the direct system in which it operates and general environment (78). The direct system consists of other components with which the initiative interacts, e.g., actors and stakeholders with various relationships. The general environment consists of less specific elements that might affect learning like economic, technological, sociocultural, and other factors. The questions probing for learning are taken from a tool to change organisations towards learning organisations (79) and focus on the frequency single-loop and double-loop learning occur at the level of individuals, teams, and the OH initiative, as well as how the system and broader environment support learning (Table S1 in Supplementary Material).

#### Sharing Infrastructure and Processes

In a broad sense, data and information sharing is a catalyser of knowledge generation (80). Data are often a pre-requisite for the operational gears to function. In OH initiatives, data and information are often the “raw material” that ultimately will lead to better understanding and a more inclusive and sustainable way of tackling the challenge. If managed appropriately, data and unbiased information sharing can foster trust between participants, as well as minimise misconduct in data management and reporting (81, 82). Additionally, this process can avoid duplication of data collection, ensuring an optimisation of resources (83).

A central benefit of data sharing is that the data can be analysed to a much greater extent than if only the data owner examines them. This brings benefits to the data owners themselves, as the analysis of others might lead them to further develop their knowledge on the systems the data originated from or the strengths and limitations of their datasets, as well as raising the awareness of the existence of the data in the wider community (80, 84, 85). Despite these benefits, data and information sharing often lead to barriers for establishing collaborations (86) and are hampered by confidentiality issues, time delays, and even mistrust in established collaborations. Consequently, data sharing is not as frequent as desirable, and needs to be incentivised to become a natural part of the science and governance cultures. For example, in some countries research relies on a tripartite agreement to share information and collaborate between academia, government institutions and industry, but public access to data may also be reinforced through legislation.

A frequent barrier to data procurement is the bureaucratic process to access data, particularly its complexity and duration. Moreover, fees and technical constraints may arise (87), and often too little resources are set aside to for data extraction from databases. Data accessibility and ownership are further critical factors, with data owned by collaborating parties contributing more to knowledge generation than public data or data owned by third parties. Data confidentiality may affect its sharing, as participant consent is usually collected for a specific purpose. This consent might not extend to new studies or alternative purposes, and therefore, security measures may be required to warrant confidentiality. Sharing sensitive data and information within a broader group might entail higher risks for confidentiality breaches (88). Alternatively, anonymization may reduce that risk, but may also reduce the utility of the data. Finally, it needs to be stressed that knowledge about the data origin and data collection processes is key for the quality and usefulness of stored data, and respective documentation must be available. For example, without knowledge about potential bias throughout the data generating process, it is extremely challenging to merge or combine data from multiple sectors in an OH initiative. The questions in Table S1 in Supplementary Material derive from a workshop held by NEOH on data and information sharing, in which critical aspects of data sharing were discussed. High scores are recommended for strong facilitation of sharing. The questions focus on the sharing mechanisms, available resources, data quality and accessibility, storage, and the resilience of these to change in the system.

#### OH Index and Ratio

Given the lack of current, commonly accepted benchmarks and the fact that OH initiatives are strongly context specific, it is recommended to assess them in relation to a context-specific benchmark. Hence, the evaluator should determine what the perfect situation in the given context would look like (using benchmarking data where they exist) and what proportion of this maximum is achieved with the OH initiative.

**Figure 4:**
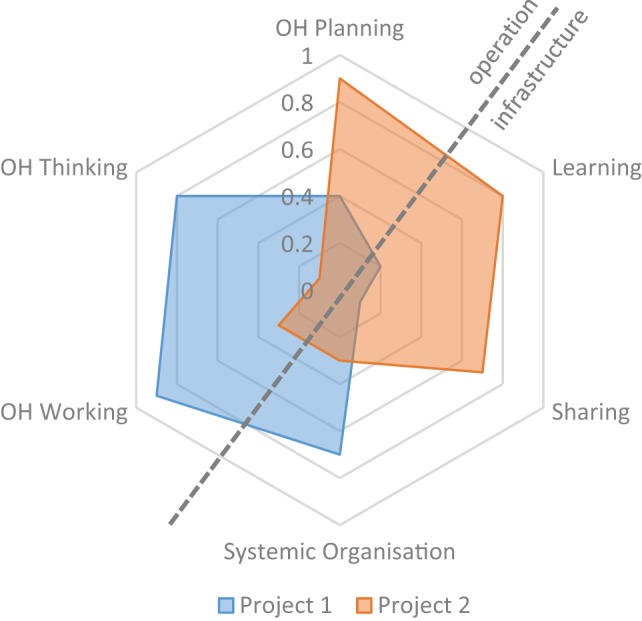
Example of the One Health (OH)-ness spider diagram for two fictive OH projects.

The aim of the OH-index (OHI) is to combine the assessments conducted in the previous sections of Element 3. To visualise the six assessments, we suggest a spider diagram (Figure 4), in which each assessment is represented by a spoke. The diagram depicts the operational aspects “OH thinking,” “OH planning,” and “OH working” opposed to the infrastructure for “learning,” “sharing,” and “systemic organisation.” Thus, the operational aspects on the top left of the diagonal are opposed to the infrastructure on the bottom right. Each spoke is scaled to cover a range of values between 0 and 1. Consequently, the plot not only illustrates the degree of integration by the surface, but it also shows the balance between the operation and the supporting means through its symmetry over the diagonal, numerically represented as the OHR.

In Figure 4, two exemplary fictive projects are depicted, an example with real data of a comparison of two OH initiatives can be found in the article by Buttigieg et al. (see text footnote 4). The fictive Project 1 depicted here has a highly developed transdisciplinary team with a very comprehensive multi-dimensional approach. However, it appears to lack learning and sharing infrastructure and has a mismatch between the responsibilities, authorities, and means which affects the transdisciplinary working and hence potentially the OH outcomes. On the other hand, Project 2 has well-developed infrastructure and well-defined tasks with sufficient funding, but does not explore the interdisciplinary space nor does it aim at serving multiple species.

The OHI corresponds to the ratio of the surface enclosed by the lines to the surface enclosed if all spokes were equal to 1 (a detailed derivation is provided in Data Sheet S1 in Supplementary Material). Thus, the OHI is

(1) $$\text{OHI}=\frac{\left\{({\text{Sc}}_{\text{P}}\times {\text{Sc}}_{\text{L}})+({\text{Sc}}_{\text{L}}\times {\text{Sc}}_{\text{P}})+({\text{Sc}}_{\text{S}}\times {\text{Sc}}_{\text{L}})+({\text{Sc}}_{\text{O}}\times {\text{Sc}}_{\text{S}})+({\text{Sc}}_{\text{W}}\times {\text{Sc}}_{\text{O}})+({\text{Sc}}_{\text{T}}\times {\text{Sc}}_{\text{W}})\right\}}{6}$$

where Sc_P_ is the score obtained in OH planning, Sc_L_ is the score obtained in learning infrastructure, Sc_S_ is the score from sharing infrastructure, Sc_O_ is the score from systemic organisation, Sc_W_ is the score from OH working, and Sc_T_ is the score from OH thinking.

The OH-ratio (OHR) is the relation of the surface covered in the top left of the diagonal to the one in the lower right (a detailed derivation is provided in Data Sheet S1 in Supplementary Material). To compute the OHR, the surface of the top left surface (SUR_operation_) is calculated

(2) $${\text{SUR}}_{\text{Operations}}=\frac{\sqrt{3}}{4}\{\left(\frac{{\text{Sc}}_{\text{O}}\times {\text{Sc}}_{\text{W}}^{2}}{{\text{Sc}}_{\text{O}}+{\text{Sc}}_{\text{W}}})+({\text{Sc}}_{\text{W}}\times {\text{Sc}}_{\text{T}}\right)+({\text{Sc}}_{\text{T}}\times {\text{Sc}}_{\text{P}})+\left(\frac{{\text{Sc}}_{\text{P}}^{2}\times {\text{Sc}}_{\text{L}}}{{\text{Sc}}_{\text{P}}+{\text{Sc}}_{\text{L}}}\right)\}$$

and divided by the surface of the lower right (SUR_infrastructure_)

(3) $${\text{SUR}}_{\text{Infrastructure}}=\frac{\sqrt{3}}{4}\{\left(\frac{{\text{Sc}}_{\text{P}}\times {\text{Sc}}_{\text{L}}^{2}}{{\text{Sc}}_{\text{P}}+{\text{Sc}}_{\text{L}}})+({\text{Sc}}_{\text{L}}\times {\text{Sc}}_{\text{S}}\right)+({\text{Sc}}_{\text{S}}\times {\text{Sc}}_{\text{O}})+\left(\frac{{\text{Sc}}_{\text{O}}^{2}\times {\text{Sc}}_{\text{W}}}{{\text{Sc}}_{\text{O}}+{\text{Sc}}_{\text{W}}}\right)\}$$

resulting in the following equation:

(4) $$\text{OHR}=\frac{\left({\text{Sc}}_{\text{O}}\times {\text{Sc}}_{\text{W}}^{2}/\left({\text{Sc}}_{\text{O}}+{\text{Sc}}_{\text{W}}\right))+({\text{Sc}}_{\text{W}}\times {\text{Sc}}_{\text{T}})+(\text{ScT}\times {\text{Sc}}_{\text{P}})+({\text{Sc}}_{\text{P}}^{2}\times {\text{Sc}}_{\text{L}}/\left({\text{Sc}}_{\text{P}}+{\text{Sc}}_{\text{L}}\right)\right)}{\left({\text{Sc}}_{\text{P}}\times {\text{Sc}}_{\text{L}}^{2}/\left({\text{Sc}}_{\text{P}}+{\text{Sc}}_{\text{L}}\right))+({\text{Sc}}_{\text{L}}\times {\text{Sc}}_{\text{S}})+({\text{Sc}}_{\text{S}}\times {\text{Sc}}_{\text{O}})+({\text{Sc}}_{\text{O}}^{2}\times {\text{Sc}}_{\text{W}}/\left({\text{Sc}}_{\text{O}}+{\text{Sc}}_{\text{W}}\right)\right)}$$

## Discussion

This new evaluation framework for OH relies on a systems approach to characterise OH (7) and elaborate guidance for its evaluation. Although several of the identified approaches and methods were established and used previously, their combination in the context of OH is new. Moreover, several modifications and enhancements were made to take into account OH specific characteristics and provide a foundation for comparison across different initiatives and the generation of new insights into the implementation of OH initiatives. The systems approach to evaluation presented here does not resolve the problem of delimitation, partiality, and bias, but the framework helps to address these factors explicitly. It also shows that the evaluator(s) is (are) part of the system of which they try to gain an understanding, as much as an OH initiative is not external to the system it tries to affect. This is particularly important when considering stakeholder perspectives, because the relationship between the evaluator(s) and the informant has an influence on the content of the feedback. Consequently, the framework formalises reflections on system dynamics and includes emerging properties in all elements. Further, it consolidates thinking, planning, working, sharing, learning, and systemic organisation in a single OHI and OHR. However, these aspects may also be investigated separately for specific circumstances. Like many systems approaches the implementation of the NEOH framework is limited by resources, but also by political and managerial endorsement. Constructive use of the evaluation framework presented demands advanced leadership skills and a facilitating learning environment. The scope of the evaluation and delimitation of the system are pivotal for the outcome of the evaluation and it is eminently important to declare how these choices impact on the results. In analogy to systems thinking in public health the concept relies critically on multi-stakeholder endorsement (89) and is vulnerable to misconceptions and misapplications (90).

Finally, care must be taken not to prejudge that a higher OHI would mean a “better” OH initiative. The authors hypothesise that there may be an optimal range of values for the OHI, outside which too little or too strong integration may hamper implementation at both ends. Also, the optimal OHR remains to be identified. As outlined earlier, the case studies reported in the present Frontiers special topic (24–26, 27), show practical applications of the evaluation framework for a variety of contexts and in different types of OH initiatives. They also provide first data on OHI and OHR, which is an important step towards their validation and the creation of a dataset for future benchmarking. Importantly, they highlighted that the qualitative evaluations are equally important to understanding the context-relevant shortcomings and strengths of the individual initiatives. However, qualitative evaluations are more difficult to compare in a meta-study compared with quantitative data due to their heterogeneity in findings. Despite this limitation, the case studies provide the foundation to improve the framework further and validate it, as they highlight ambiguities and shortcomings in practical application.

## Author Contributions

The manuscript bases on extracts of an iterative process for the production of the handbook for evaluation of One Health of the COST Action TD1404 “Network for Evaluation of One Health.” The working group preparing the handbook was co-lead by SR, while the COST Action was chaired by BH. SR prepared the manuscript, which was revised primarily by LN and BH. Conceptual and text contributions were made by SB, MS, MA, MC, TE, IC, EB, MR, KQ, and MB.

## Conflict of Interest Statement

The reviewer MN and handling Editor declared their shared affiliation.

## Footnotes

^1^http://neoh.onehealthglobal.net.

^2^https://www.frontiersin.org/research-topics/5479.

^3^Léger A, Stärk K, Rushton J, Nielsen LR. A one health evaluation of the University of Copenhagen Research Centre for Control of Antibiotic Resistance. *Front Vet Sci* (2017). (under review in this Research Topic).

^4^Buttigieg SC, Savic S, Cauchi D, Lautier E, Canali M, Aragrande M. Comparing the control and eradication of brucellosis in Malta and Serbia: a one health evaluation. *Front Vet Sci* (2017). (under review in this Research Topic).

^5^Muñoz-Prieto A, Nielsen LR, Martinez-Subiela S, Mazeikiene J, Lopez Jornet PL, Savic S, et al. Evaluation of one health operations and supporting infrastructure in a questionnaire-based study of obesity among dogs and their owners in 11 European countries. *Front Vet Sci* (2017). (under review in this Research Topic).

^6^Fonseca AG, Torgal J, De Meneghi D, Gabriel S, Coelho AC, Vilhena M. One health ness evaluation of the surveillance design of Taeniasis and Cysticercosis in Portugal (a neglected disease in Europe). *Front Public Health* (2017). (under review in this Research Topic).

## References

[1] Food and Agriculture Organization. In: Raney T, editor. The State of Food and Agriculture. Rome, Italy: Food and Agriculture Organization of the United Nations (2013). Available from: http://www.fao.org/docrep/018/i3300e/i3300e00.htm

[2] Jones KE Patel NG Levy MA Storeygard A Balk D Gittleman JL Global trends in emerging infectious diseases. Nature (2008) 451:990–4. 10.1038/nature06536 18288193 PMC5960580

[3] Pfeiffer DU. From risk analysis to risk governance—adapting to an ever more complex future. Vet Ital (2014) 50:169–76. 10.12834/VetIt.313.1220.3 25273958

[4] Whitmee S Haines A Beyrer C Boltz F Capon AG de Souza Dias BF Safeguarding human health in the anthropocene epoch: report of the Rockefeller Foundation–Lancet Commission on planetary health. Lancet (2015) 6736:1–56. 10.1016/S0140-6736(15)60901-1 26188744

[5] Romanelli C Cooper D Campbell-Ledrum D Maiero M Karesh WB Danny H Connecting Global Priorities: Biodiversity and Human Health—A State of Knowledge Review. Geneva (2015). Available from: https://www.cbd.int/health/SOK-biodiversity-en.pdf

[6] Zinsstag J Schelling E Waltner-Toews D Tanner M From “one medicine” to “one health” and systemic approaches to health and well-being. Prev Vet Med (2011) 101:148–56. 10.1016/j.prevetmed.2010.07.003 20832879 PMC3145159

[7] Rüegg SR McMahon BJ Häsler B Esposito R Rosenbaum Nielsen L Ifejika Speranza C A blueprint to evaluate one health. Front Public Health (2017) 5:20 10.3389/fpubh.2017.00020 28261580 PMC5311072

[8] Stokols D Hall KL Vogel AL Transdisciplinary public health: definitions, core characteristics and strategies for success. In: Haire-Joshu D McBride TD, editors. Transdisciplinary Public Health: Reserach, Methods and Practice. San Francisco: Jossey-Bass (2013). p. 3–30.

[9] Ledford BYH How to solve the world’s biggest problems. Nature (2015) 525:308–11. 10.1038/525308a 26381968

[10] Rabinowitz PM Kock R Kachani M Kunkel R Thomas J Gilbert J Toward proof of concept of a one health approach to disease prediction and control. Emerg Infect Dis (2013) 19:e130265. 10.3201/eid1912.130265 24295136 PMC3840882

[11] Stokols D Fuqua J Gress J Harvey R Phillips K Baezconde-Garbanati L Evaluating transdisciplinary science. Nicotine Tob Res (2003) 5:21–39. 10.1080/14622200310001625555 14668085

[12] Lattuca LR Knight DB Bergom IM Developing a measure of interdisciplinary competence for engineers. Conference Proceedings 2012 of the American Society for Engineering Education p. 1–19. Available from: https://peer.asee.org/21173

[13] Anderson V Johnson L Systems Thinking Basics: From Concepts to Causal Loops. Acton, USA: Leverage Networks Inc (1997).

[14] Whitehead NP Scherer WT Smith MC Systems thinking about systems thinking. IEEE Syst J (2015) 9:1117–28. 10.1109/JSYST.2014.2332494

[15] Ostrom E. A general framework for analyzing sustainability of social-ecological systems. Science (2009) 325:419–22. 10.1126/science.1172133 19628857

[16] WHO. In: de Savigny D Taghreed A, editors. Systems Thinking for Health Systems Strengthening. Geneva: World Health Organisation (2009). Available from: http://apps.who.int/iris/bitstream/10665/44204/1/9789241563895_eng.pdf

[17] Anonymous. Interactive Terminology for Europe. (1999). Available from: http://iate.europa.eu

[18] Pumain D Pavé A Venant FA Verdier N Victorri B West GB Hierarchy in Natural and Social Sciences. 1st ed Dorderecht, The Netherlands: Springer (2006).

[19] Brown A-M Differences between the Theory of Change and the Logic Model. (2016). Available from: https://www.annmurraybrown.com/single-post/2016/03/20/Theory-of-Change-vsThe-Logic-Model-Never-Be-Confused-Again

[20] OECD. Glossary of Key Terms in Evaluation and Results Based Management. (2010). 38 p. Available from: http://www.oecd.org/dac/evaluation/2754804.pdf

[21] Haxton E Sinigoj S Rivière-Cinnamond A The network for evaluation of one health: evidence-based added value of one health. Infect Ecol Epidemiol (2015) 5:28164 10.3402/iee.v5.28164 26426072 PMC4590410

[22] Trochim WM Cabrera DA Milstein B Gallagher RS Leischow SJ. Practical challenges of systems thinking and modeling in public health. Am J Public Health (2006) 96:538–46. 10.2105/AJPH.2005.066001 16449581 PMC1470516

[23] Garcia JR Zazueta A Going beyond mixed methods to mixed approaches: a systems perspective for asking the right questions. IDS Bull (2015) 46:30–43. 10.1111/1759-5436.12119

[24] Paternoster G Tomassone L Tamba M Chiari M Lavazza A Piazzi M The degree of one health implementation in the West Nile virus integrated surveillance in Northern Italy, 2016. Front Public Health (2017) 5:236. 10.3389/fpubh.2017.00236 28929098 PMC5591825

[25] Radeski M O’Shea H De Meneghi D Ilieski V Positioning animal welfare in the one health concept through evaluation of an Animal Welfare Center in the Faculty of Veterinary Medicine, Skopje, Macedonia. Front Vet (2017) 4:238 10.3389/fvets.2017.00238 PMC5767597 29376062

[26] Laing G Aragrande M Canali M Savic S De Meneghi D Control of cattle ticks and tick-borne diseases by acaricide in Southern Province of Zambia: a retrospective evaluation of animal health measures according to current one health concepts. Front Public Health (2017). 10.3389/fpubh.2018.00045 PMC5881173 29637063

[27] Hanin MCE Queenan K Savic S Rüegg SR Häsler B A one health evaluation of the Southern African Centre for Infectious Disease Surveillance. Front Vet Sci (2017). 10.3389/fvets.2018.00033 PMC5864892 29616227

[28] Williams B Using Systems Concepts in Evaluation Desing: A Workbook. 1st ed Kyoto (2016). Available from: http://www.bobwilliams.co.nz

[29] Bunge M Levels: a semantical preliminary. Rev Metaphys (1960) 13:396–406. 10.1177/0306312708091929

[30] Lerner H Berg C. The concept of health in one health and some practical implications for research and education: what is one health? Infect Ecol Epidemiol (2015) 5:25300. 10.3402/iee.v5.25300 25660757 PMC4320999

[31] Boriani E Esposito R Frazzoli C Fantke P Hald T Rüegg SR Framework to Define Structure and Boundaries of Complex Health Intervention Systems: The ALERT Project. Front Public Heal (2017) 5 10.3389/fpubh.2017.00182 PMC5532392 28804707

[32] Craig P Dieppe P Macintyre S Michie S Nazareth I Petticrew M Developing and evaluating complex interventions: the new medical research council guidance. Int J Nurs Stud (2013) 50:587–92. 10.1016/j.ijnurstu.2012.09.010 23159157

[33] Lang DJ Wiek A Bergmann M Stauffacher M Martens P Moll P Transdisciplinary research in sustainability science: practice, principles, and challenges. Sustain Sci (2012) 7:25–43. 10.1007/s11625-011-0149-x

[34] Taplin DH Clark H Collins E Colby DC Theory of Change: Technical Papers: A Series of Papers to Support Development of Theories of Change Based on Practice in the Field. (2013). 23 p. Available from: http://www.theoryofchange.org/wp-content/uploads/toco_library/pdf/ToC-Tech-Papers.pdf

[35] INTRAC. Outputs, Outcome and Impact. (2015). Available from: https://www.intrac.org/wpcms/wp-content/uploads/2016/06/Monitoring-and-Evaluation-Series-Outcomes-Outputs-and-Impact-7.pdf

[36] Deprez S The 5 Key Assumptions of Outcome Mapping. (2014). Available from: https://www.outcomemapping.ca/nuggets/the-5-key-assumptions-of-outcome-mapping

[37] Häsler B Cornelson L Bennani H Rushton J. A review of the metrics for one health benefits. Rev Sci Tech (2014) 33:453–64. 25707176 10.20506/rst.33.2.2294

[38] Baum SE Machalaba C Daszak P Salerno RH Karesh WB. Evaluating one health: are we demonstrating effectiveness? One Health (2016) 3:5–10. 10.1016/j.onehlt.2016.10.004 28616496 PMC5458598

[39] Falzon L Lechner I Chantziaras I Collineau L Courcoul A Filippitzi M The Quantitative Outcomes of a “One Health” Approach to Study Global Health Issues: A Systematic Review. EcoHealth (2018). 10.1007/s10393-017-1310-5 PMC6003973 29330676

[40] Strang V McLeish T Evaluating Interdisciplinary Research: A Practical Guide. Report (2015). p. 1–20. Available from: https://www.dur.ac.uk/ias/news/?itemno=25309

[41] Ingram J White R Inaugural lecture—food systems: challenges, concepts and communities. Lecture (2015). Available from: http://ifstal.ouce.ox.ac.uk/news-and-events/recent-events/

[42] Lélé S Norgaard RB Practicing interdisciplinarity. Bioscience (2005) 55:967 10.1641/0006-3568(2005)055[0967:PI]2.0.CO;2

[43] Hirsch Hadorn G Hoffmann-Riem H Biber-Klemm D Grossenbacher-Mansuy S Joye W Pohl C, editors. Handbook of Transdisciplinary Research. 1st ed Springer (2008). Available from: https://link.springer.com/book/10.1007/978-1-4020-6699-3

[44] Reynolds M (Breaking) the iron triangle of evaluation. IDS Bull (2015) 46:71–86. 10.1111/1759-5436.12122

[45] Fath BD Dean CA Katzmair H Navigating the adaptive cycle: an approach to managing the resilience of social systems. Ecol Soc (2015) 20:art24 10.5751/ES-07467-200224

[46] Wallace RG Wallace R, editors. Neoliberal Ebola. Cham: Springer (2016).

[47] Mowles C Complex, but not quite complex enough: the turn to the complexity sciences in evaluation scholarship. Evaluation (2014) 20:160–75. 10.1177/1356389014527885

[48] Chigas D Ehlinger T Befani B Docherty J Michaels J Smith R Non-linear Impact Assessment: Challenges, Approaches and Tools. Honolulu, Hawaii (2014).

[49] Befani B Ramalingam B Stern E Introduction—towards systemic approaches to evaluation and impact. IDS Bull (2015) 46:1–6. 10.1111/1759-5436.12116

[50] Britt H Discussion Note: Complexity-Aware Monitoring. Washington, DC (2016). Available from: https://usaidlearninglab.org/library/complexity-aware-monitoring-discussion-note-brief

[51] Moore G Audrey S Barker M Bond L Process Evaluation of Complex Interventions. (2014). Available from: http://decipher.uk.net/wp-content/uploads/2014/11/MRC-PHSRN-Process-evaluation-guidance.pdf 10.1136/bmj.h1258 PMC4366184 25791983

[52] Moore GF Audrey S Barker M Bond L Bonell C Hardeman W Process evaluation of complex interventions: medical research council guidance. BMJ (2015) 350:h1258 10.1136/bmj.h1258 25791983 PMC4366184

[53] Meadows DH In: Wright D, editor. Thinking in Systems—A Primer. Chelsea Green Publishing Co (2008). Available from: https://books.google.ch/books?hl=en&lr=&id=CpbLAgAAQBAJ&oi=fnd&pg=PR9&dq=Donella+Maedows+thinking+in+systems&ots=LypcqbwER1&sig=f-9XJp4hf9FLf6IWgQkMEXg6GI8&redir_esc=y#v=onepage&q=Donella Maedows thinking in systems&f=false

[54] Whitehead NP Scherer WT Quantifying the quality of a systems approach. 2015 Annual IEEE Systems Conference (SysCon) Proceedings (IEEE). Vancouver, BC (2015). p. 44–9.

[55] Gunderson LH Cosens B Garmestani AS. Adaptive governance of riverine and wetland ecosystem goods and services. J Environ Manag (2016) 183:353–60. 10.1016/j.jenvman.2016.05.024 27206806 PMC7313720

[56] In praise of soft science. Nature (2005) 435:1003 10.1038/4351003a 15973363

[57] Hadorn Hirsch G Hoffmann-Riem H Biber-Klemm S Grossenbacher-Mansuy W Joye D Pohl C Handbook of Transdisciplinary Research. Springer (2007). Available from: http://www.springer.com/gb/book/9781402066986

[58] Nikitina S Pathways of interdisciplinary cognition. Cogn Instr (2005) 23:389–425. 10.1207/s1532690xci2303_3

[59] Nancarrow SA Booth A Ariss S Smith T Enderby P Roots A. Ten principles of good interdisciplinary team work. Hum Resour Health (2013) 11:19. 10.1186/1478-4491-11-19 23663329 PMC3662612

[60] Aragrande M Canali M An operational tool to enhance one health interdisciplinarity. Proceedings 3rd GRF One Health Summit.

[61] Thygeson M Morrissey L Ulstad V. Adaptive leadership and the practice of medicine: a complexity-based approach to reframing the doctor-patient relationship. J Eval Clin Pract (2010) 16:1009–15. 10.1111/j.1365-2753.2010.01533.x 20846289

[62] Yukl G Effective leadership behavior: what we know and what questions need more attention. Acad Manag Perspect (2012) 26:66–85. 10.5465/amp.2012.0088

[63] Rooke D Torbert WR Seven transformations of leadership. Harv Bus Rev (2005). Available from: https://hbr.org/2005/04/seven-transformations-of-leadership::x00023::15807040

[64] Scott L Caress A-L. Shared governance and shared leadership: meeting the challenges of implementation. J Nurs Manag (2005) 13:4–12. 10.1111/j.1365-2834.2004.00455.x 15613089

[65] Houghton JD Pearce CL Manz CC Courtright S Stewart GL Sharing is caring: toward a model of proactive caring through shared leadership. Hum Resour Manag Rev (2015) 25:313–27. 10.1016/j.hrmr.2014.12.001

[66] Fiol MC Lyles MA Organizational learning. Acad Manag Rev (1985) 10:803–13. 10.5465/AMR.1985.4279103

[67] Tsang EWK Organizational learning and the learning organization: a dichotomy between descriptive and prescriptive research. Hum Relat (1997) 50:73–89. 10.1177/001872679705000104

[68] Levitt B March JG Organizational learning. Ann Rev Soc (1988) 14:319–40. 10.1146/annurev.so.14.080188.001535

[69] Argyris C On Organizational Learning. 2nd ed Oxford, UK: Wiley-Blackwell (1999).

[70] Giesecke J McNeil B Transitioning to the learning organization. Libr Trends (2004) 53:54.

[71] Redding JC Catalanello RF Strategic Readiness: The Making of the Learning Organization. Jossey-Bass (1994).

[72] Gould N Becoming a learning organisation: a social work example. Soc Work Educ (2000) 19:585–96. 10.1080/02615470020002317

[73] Watkins KE Marsick VJ Sculpting the Learning Organization: Lessons in the Art and Science of Systemic Change. 1st ed San Francisco, CA: Jossey-Bass (1993).

[74] Guns B The Faster Learning Organization. 1st ed Jossey-Bass (1998).

[75] Garvin DA Learning in Action: A Guide to Putting the Learning Organization to Work. Boston, MA: Harward Business Press (2000).

[76] Huysman M Balancing biases: a critical review of the literature on organizational learning. In: Easterby-Smith M Araujo L Burgoyne J editors. Organizational Learning and the Learning Organization: Developments in Theory and Practice. London: SAGE (1999). p. 59–74.

[77] Santa M Learning Organisation Review – a “Good” Theory Perspective. The Learning Organization (2015) 22(5):242–70. 10.1108/TLO-12-2014-0067

[78] Santa M Framework for Multivariate Continuous Transformation Towards Learning Organization [Ph.D. thesis]. Paris: Pantheon-Sorbonne University (2014).

[79] Santa M Chapter 5 the Learning Organization Atlas Framework [Ph.D. thesis]. (2001).

[80] Piwowar HA Day RS Fridsma DB. Sharing detailed research data is associated with increased citation rate. PLoS One (2007) 2:e308. 10.1371/journal.pone.0000308 17375194 PMC1817752

[81] Schelling E Zinsstag J Transdisciplinary research and one health. In: Zinsstag J Schelling E Waltner-Toews D Whittaker M Tanner M, editors. One Health: The Theory and Practice of Integrated Health Approaches. Oxfordshire: CABI (2015). p. 366–73. Available from: www.cabi.org

[82] Walter AI Helgenberger S Wiek A Scholz RW. Measuring societal effects of transdisciplinary research projects: design and application of an evaluation method. Eval Program Plann (2007) 30:325–38. 10.1016/j.evalprogplan.2007.08.002 17904636

[83] Tenopir C Allard S Douglass K Aydinoglu AU Wu L Read E Data sharing by scientists: practices and perceptions. PLoS One (2011) 6:e21101. 10.1371/journal.pone.0021101 21738610 PMC3126798

[84] Piwowar HA Chapman WW. Public sharing of research datasets: a pilot study of associations. J Informetr (2010) 4:148–56. 10.1016/j.joi.2009.11.010 21339841 PMC3039489

[85] Piwowar HA Becich MJ Bilofsky H Crowley RS Towards a data sharing culture: recommendations for leadership from academic health centers. PLoS Med (2008) 5:1315–9. 10.1371/journal.pmed.0050183 PMC2528049 18767901

[86] Chokshi DA Parker M Kwiatkowski DP. Data sharing and intellectual property in a genomic epidemiology network: policies for large-scale research collaboration. Bull World Health Organ (2006) 84:382–7. 10.2471/BLT.06.029843 16710548 PMC2627357

[87] Houe H Gardner IA Nielsen LR. Use of information on disease diagnoses from databases for animal health economic, welfare and food safety purposes: strengths and limitations of recordings. Acta Vet Scand (2011) 53(Suppl 1):S7. 10.1186/1751-0147-53-S1-S7 21999520 PMC3194126

[88] Borgman CL Scholarship in the Digital Age: Information, Infrastructure, and the Internet. Cambridge, MA: MIT Press (2010).

[89] El-Jardali F Adam T Ataya N Jamal D Jaafar M Constraints to applying systems thinking concepts in health systems: a regional perspective from surveying stakeholders in Eastern Mediterranean countries. Int J Health Policy Manag (2014) 3:399–407. 10.15171/ijhpm.2014.124 25489598 PMC4258892

[90] Canyon DV Systems thinking: basic constructs, application challenges, misuse in health, and how public health leaders can pave the way forward. Hawaii J Med Public Health (2013) 72:440–4. 24377080 PMC3872923

