# Supplementary material for: A Systems Approach to Evaluate One Health Initiatives
Source: Front Vet Sci. 2018 Mar 9;5:23. doi: 10.3389/fvets.2018.00023 (PMC5854661; doi:10.3389/fvets.2018.00023)
Supplement: Supplementary file 1 [file Data_Sheet_1.docx]

# Calculation of the One Health Index and Ratio

Supplementary material to the manuscript “"A systems approach to evaluate One Health" by Rüegg et al. 2017.

Detailed derivation of the equation is provided by Simon Rüegg and Miroslav Radeski.

1. One Health Index: the surface of the hexagon

OH Thinking

Sharing

Learning

OH Planning

OH Working

Systemic Organisation

ScT

H

ScL

ScP

ScS

ScO

ScW

The angle between two spokes (Sc) is = 2Π/6 = Π/3 = 60°; $\sin60^{\circ}=\frac{\sqrt{3}}{2}$;

$\sin\frac{\pi}{3}=\frac{H}{ScT} ; H=ScT\times\sin\frac{\pi}{3}$; $sector=\frac{ScP\times H}{2}=\frac{ScP\times ScT\times\sin\frac{\pi}{3}}{2}=\frac{\sqrt{3}}{4}\times ScP\times ScT$;

$$Surface of hexagon=\sum all sectors$$

$$\sum sect.=\frac{\sqrt{3}}{4}\left\{ \left( ScP\times ScT \right)+\left( ScL\times ScP \right)+\left( ScS\times ScL \right)+\left( ScO\times ScS \right)+\left( ScW\times ScO \right)+\left( ScT\times ScW \right) \right\}$$

The OH-index is the surface covered by the evaluated initiative over the total achievable surface:

$$OHI=\frac{\frac{\sqrt{3}}{4}\left\{ \left( ScP\times ScT \right)+\left( ScL\times ScP \right)+\left( ScS\times ScL \right)+\left( ScO\times ScS \right)+\left( ScW\times ScO \right)+\left( ScT\times ScW \right) \right\}}{\frac{\sqrt{3}}{4}\left\{ 1+1+1+1+1+1 \right\}}$$

$$OHI=\frac{\left\{ \left( ScP\times ScT \right)+\left( ScL\times ScP \right)+\left( ScS\times ScL \right)+\left( ScO\times ScS \right)+\left( ScW\times ScO \right)+\left( ScT\times ScW \right) \right\}}{6}$$

(1)

1. The One Health Ratio:

Surface of top left section (operations)

OH Thinking

Sharing

Learning

OH Planning

OH Working

Systemic Organisation

ScT

H

ScL

ScP

ScS

ScO

ScW

Tb

Ta

The surface of the area covered operations is the sum of the surfaces of the four triangles to the top left of the diagonal, the area for infrastructure the sum of the surfaces of the four triangles to the lower right.

To determine the two parts of the triangle between ScP and ScL:

$$surface of first triangle:T_{\mathrm{PTa}}=\frac{1}{2}\mathrm{ScP}\times Ta\times\sin30^{\circ}$$

$surface of second triangel: T_{\mathrm{LTa}}=\frac{1}{2}ScL\times Ta\times\sin30^{\circ}$, thus

$\frac{1}{2}\mathrm{ScP}\times ScL\times\sin60^{\circ}=\frac{1}{2}\mathrm{ScP}\times Ta\times\sin30^{\circ}+ \frac{1}{2}ScL\times Ta\times\sin30^{\circ}$;

with $\sin30^{\circ}=\frac{1}{2}\mathrm{and}\sin60^{\circ}=\frac{\sqrt{3}}{2}$

$\frac{\sqrt{3}}{2}\mathrm{ScP}\times ScL=\frac{1}{2}\mathrm{ScP}\times Ta+ \frac{1}{2}ScL\times Ta=\frac{1}{2}Ta\times\left( ScP+ScL \right)$

$$Ta=\sqrt{3}\frac{\mathrm{ScP}\times\mathrm{ScL}}{ScP+ScL}$$

In analogy $Tb=\sqrt{3}\frac{\mathrm{ScO}\times\mathrm{ScW}}{ScO+ScW}$

The SUR_Operations_ (top left surface):

$$\mathrm{SUR}_{\mathrm{Operations}}=\frac{\mathrm{Tb}\times ScW\times\sin30^{\circ}}{2}+\frac{\mathrm{ScW}\times ScT\times\sin60^{\circ}}{2}+\frac{\mathrm{ScT}\times ScP\times\sin60^{\circ}}{2}+\frac{ScP\times Ta\times\sin30^{\circ}}{2}=\frac{1}{4}\left\{ \left( \sqrt{3}\frac{\mathrm{ScO}\times\mathrm{ScW}}{ScO+ScW}\times\mathrm{ScW} \right)+\left( \mathrm{ScW}\times ScT\times\sqrt{3} \right)+\left( \mathrm{ScT}\times ScP\times\sqrt{3} \right)+\left( \sqrt{3}\frac{\mathrm{ScP}\times\mathrm{ScL}}{ScP+ScL} \times ScP \right) \right\}=\frac{\sqrt{3}}{4}\left\{ \left( \frac{\mathrm{ScO}\times\mathrm{ScW}^{2}}{ScO+ScW} \right)+\left( \mathrm{ScW}\times\mathrm{ScT} \right)+\left( \mathrm{ScT}\times\mathrm{ScP} \right)+\left( \frac{\mathrm{ScP}^{2}\times\mathrm{ScL}}{ScP+ScL} \right) \right\}$$

The SUR_Infrastructure_ (bottom right surface):

$$\mathrm{SUR}_{\mathrm{Infrastructure}}=\frac{\mathrm{Ta}\times ScL\times\sin30^{\circ}}{2}+\frac{\mathrm{ScL}\times ScS\times\sin60^{\circ}}{2}+\frac{\mathrm{ScS}\times ScO\times\sin60^{\circ}}{2}+\frac{ScO\times Tb\times\sin30^{\circ}}{2}=\frac{1}{4}\left\{ \left( \sqrt{3}\frac{\mathrm{ScP}\times\mathrm{ScL}}{ScP+ScL}\times\mathrm{ScL} \right)+\left( \mathrm{ScL}\times ScS\times\sqrt{3} \right)+\left( \mathrm{ScS}\times ScO\times\sqrt{3} \right)+\left( \sqrt{3}\frac{\mathrm{ScO}\times\mathrm{ScW}}{ScO+ScW} \times ScO \right) \right\}=\frac{\sqrt{3}}{4}\left\{ \left( \frac{\mathrm{ScP}\times\mathrm{ScL}^{2}}{ScP+ScL} \right)+\left( \mathrm{ScL}\times\mathrm{ScS} \right)+\left( \mathrm{ScS}\times\mathrm{ScO} \right)+\left( \frac{\mathrm{ScO}^{2}\times\mathrm{ScW}}{ScO+ScW} \right) \right\}$$

Ratio

$OHR=\frac{\left( \frac{\mathrm{ScO}\times\mathrm{ScW}^{2}}{ScO+ScW} \right)+\left( \mathrm{ScW}\times\mathrm{ScT} \right)+\left( \mathrm{ScT}\times\mathrm{ScP} \right)+\left( \frac{\mathrm{ScP}^{2}\times\mathrm{ScL}}{ScP+ScL} \right)}{\left( \frac{\mathrm{ScP}\times\mathrm{ScL}^{2}}{ScP+ScL} \right)+\left( \mathrm{ScL}\times\mathrm{ScS} \right)+\left( \mathrm{ScS}\times\mathrm{ScO} \right)+\left( \frac{\mathrm{ScO}^{2}\times\mathrm{ScW}}{ScO+ScW} \right)}$ (2)
